# Mechanism of triptolide regulating proliferation and apoptosis of hepatoma cells by inhibiting JAK/STAT pathway

**DOI:** 10.1515/biol-2022-1018

**Published:** 2025-01-27

**Authors:** Guanglian Wang, Zhenxin Zhu, Zhengang Sun, Zhiqi Yang

**Affiliations:** Department of Hepatobiliary Pancreatic and Splenic Surgery, Jingzhou Central Hospital, Jingzhou 434020, Hubei, China

**Keywords:** triptolide, JAK/STAT pathway, hepatoma cells, proliferation, apoptosis

## Abstract

This study was to analyze the effect of triptolide (TPL) on proliferation, apoptosis, and the relationship between TPL and the Janus kinase/signal transducer and activator of transcription signaling pathway in hepatoma cells. HepG2 cell line was selected as the experimental object and divided into control, low-dose TPL, medium-dose TPL, and high-dose TPL group. The control group did not receive any drug treatment, while the low, medium, and high-dose groups were treated with TPL at concentrations of 0.02, 0.05, and 0.10 μM, respectively. 3-(4,5-Dimethylthiazol-2-yl)-2,5-diphenyl tetrazolium bromide, flow cytometry, and western blot were used to detach the TPL effect and mechanism. The cell proliferation inhibition rate in each dose group of TPL was lower than that in the control group, and the inhibition rate of cell proliferation increased with the increase of TPL dose (*P* < 0.05). The apoptosis rate of TPL in each dose group was higher than that in the control group, and the apoptosis rate increased with the increase of TPL dose (*P* < 0.05). The expression of phosphorylated Janus kinase 1 (p-JAK1) and phosphorylated signal transducer and activator of transcription 3 (p-STAT3) protein in cells of each dose group of TPL was lower than that in the control group, and the expression of p-JAK1 and p-STAT3 protein decreased with the increase of TPL dose (*P* < 0.05). The apoptosis rate of 10 ng/mL transforming growth factor-beta + high-dose group was reduced than that in the high-dose group, and the expression of p-JAK1 and p-STAT3 protein was higher than that in the high-dose group (*P* < 0.05). The activity of B-cell lymphoma/leukemia-2-associated X protein (Bax) protein and cysteine aspartic acid protease (Caspase)-3/9 in TPL cells at each dose was raised than that in the control group, and the expression of B-cell lymphoma/leukemia-2 (Bcl-2) protein was decreased than that in the control group. With the increase of TPL dose, the activity of Bax protein and Caspase-3/9 increased, and the Bcl-2 protein decreased (*P* < 0.05). As an anti-liver cancer agent, TPL inhibits the proliferation of hepatocellular carcinoma cells and promotes apoptosis. The mechanism may involve inhibiting Janus kinase 1/signal transducer and activator of transcription 3 pathway and activation of apoptosis-related pathways.

## 1 Introduction

In the world, hepatocellular carcinoma (HCC) is the second leading cause of cancer-related death [1]. Every year, 600,000–800,000 new HCC cases are reported worldwide [2]. Surgical resection and liver transplantation are the most appropriate treatments for HCC early stages, while in the middle stages, local treatment, such as radiotherapy [3], polykinase inhibitors such as sorafenib, ravatinib, regfinib, and carbotinib were used as first-line therapy. Despite the clinical benefits of these drugs, patient survival and outcomes have not improved significantly [4]. Drug resistance and liver dysfunction hinder these drugs’ effectiveness. The development of better therapeutic drugs is therefore urgently needed, especially for patients with advanced HCC.

Triptolide (TPL) is a lipophilic extract and the main active compound isolated from *Tripterygium wilfordii*. In addition to immunosuppressive and anti-inflammatory properties, TPL has attracted wide attention because of its anti-tumor effect [5]. TPL has been reported to be involved in the regulation of apoptosis in a variety of cancer cells by activating various signaling pathways including the tumor suppressor p53, the Fas death and mitochondrial pathways, and the transcription of Mcl-1 [6]. TPL interfered with the activation and signaling of the Janus kinase 1/signal transducer and activator of transcription 3 (JAK1/STAT3) pathway by inhibiting key components, particularly by downregulating the phosphorylation levels of phosphorylated Janus kinase 1 (p-JAK1) and phosphorylated signal transducer and activator of transcription 3 (p-STAT3). This inhibition suppressed the proliferation of HCC cells and promoted their apoptosis. This effect may be mediated either through direct action on Janus kinase (JAK) or via other upstream regulatory factors, thereby blocking the nuclear translocation of STAT3 and its transcriptional regulation of downstream target genes. Consequently, this led to cell cycle arrest and the upregulation of apoptosis-related proteins such as B-cell lymphoma/leukemia-2-associated X protein (Bax) and cysteine aspartic acid protease (Caspase)-3/9, along with the downregulation of the anti-apoptotic protein B-cell lymphoma/leukemia-2 (Bcl-2). This mechanism not only elucidates the multifaceted anticancer properties of TPL but also provides a robust biological basis for its potential as a Janus kinase/signal transducer and activator of transcription (JAK/STAT) pathway inhibitor. Research by Reece et al. [7] demonstrated that JAK1/2 inhibitors can serve as immunomodulators for the treatment and cure of various viral infections. Yuan et al. [8] reviewed recent studies on natural products that induce apoptosis in cancer cells, summarizing pro-apoptotic mechanisms and highlighting the relevance of the JAK1/STAT3 pathway and apoptotic signaling.

However, the mechanism of TPL interaction with the JAK/STAT pathway in HCC remains unclear. The purpose of this study was to explore the TPL on proliferation and apoptosis of HCC cells, and to clarify the relationship between TPL and JAK/STAT pathway and its mechanism.

## 2 Materials and methods

### 2.1 Cell line

HCC cell line HepG2 was used in this study, which was provided by Shanghai Bohu Biotechnology Co., Ltd.

### 2.2 Methods

#### 2.2.1 Cell resuscitation

The cryopreservation tube was removed from the liquid nitrogen tank and quickly placed into a preheated water bath for shaking and thawing. Once the cells were transferred to a 15 mL centrifuge tube, the volume of culture medium was adjusted to 10 mL. The mixture was centrifuged for 5 min at 800 rpm and 4°C. The cells were then resuscitated with 3 mL of Dulbecco’s Modified Eagle Medium (DMEM) and cultured in an incubator containing 5% carbon dioxide (CO_2_) at 37°C, with the culture medium changed after the cells adhered to the dish. The lid of the petri dish was opened, and the cells were washed with phosphate-buffered saline (PBS) 1–2 times. Trypsin solution was added, and the bottom of the cell petri dish was washed gently before being placed in the incubator at 37°C for 2–3 min. The cell suspension was transferred to a new petri dish in the same proportion and placed in a CO₂ incubator. Cell growth in the petri dish was observed daily, and passage and follow-up experiments were conducted when the cell confluence reached 80%.

#### 2.2.2 Cell culture and grouping

HepG2 cells were cultured in DMEM supplemented with 10% fetal bovine serum at 37°C and 5% CO_2_ for 3 days, after which the cells were digested and passaged upon reaching 80–90% confluence. These cells were subsequently resuscitated and passed on to the third generation of stable growth. The low-dose group was treated with TPL at a concentration of 0.02 μM, the medium-dose group with 0.05 μM, and the high-dose group with 0.10 μM. The blank control group received a TPL concentration of 0.00 μM, while an additional group was treated with TPL at 0.10 μM in combination with 10 ng/mL of TGF-β1. In this study, TPL was initially dissolved in the organic solvent dimethyl sulfoxide (DMSO) before being diluted into the culture medium.

### 2.3 Observation indicators

#### 2.3.1 Detection of cell proliferation by methyl thiazolyl tetrazolium (MTT) method

Cells were processed 48 h post-transfection. They were gently washed twice with PBS to remove the old culture medium and other impurities, followed by the addition of 0.5 mL of 0.25% trypsin solution to each well to detach the cells from the culture surface. Once the cells were fully detached, the culture plate was gently tapped or the cell suspension was pipetted to achieve uniform dispersion. Subsequently, 10 μL of medium containing 5 mg/mL methyl thiazolyl tetrazolium (MTT) solution was added to each well. The cells were incubated at 37°C in a 5% CO_2_ atmosphere for 4 h. After incubation, the supernatant was carefully aspirated, and 150 μL of DMSO was added to each well as a stop solution. The culture plate was gently shaken at room temperature for 10 min to ensure uniformity of the solution. The absorbance of each well was measured at 490 nm using a microplate reader and the data were recorded and analyzed.

#### 2.3.2 Detection of apoptosis rate of cells by flow cytometry

Cells from all four groups were collected 24 h post-drug treatment and gently washed once with PBS to remove culture medium and other impurities. Trypsin was used to digest the cells, allowing them to detach from the culture surface. The cell suspension was collected and centrifuged, and the supernatant was discarded. The cells were resuspended in PBS and centrifuged again, with the supernatant discarded. The cells were fixed in 70% ice-cold ethanol to achieve a final concentration of approximately 1 × 10^6^ cells/mL and fixed for 1 h. Following fixation, the cell suspension was centrifuged at 1,500 rpm for 5 min, and the supernatant was discarded. The cells were resuspended in 0.5 mL of PBS and centrifuged again, discarding the supernatant. They were then resuspended in 0.5 mL of PBS and treated with RNase to a final concentration of 100 μg/mL. The cell suspension was incubated in a 37°C water bath for 30 min to degrade ribonucleic acid (RNA), thereby preventing interference with propidium iodide (PI) staining used for apoptosis detection. After incubation, the cell suspension was centrifuged, and the supernatant was discarded. The cells were resuspended in 0.5 mL of PBS, and PI was added to a final concentration of 50 μg/mL. The cell suspension was then incubated in the dark at 4°C for 30 min to allow sufficient PI uptake into the cell nuclei and binding to deoxyribonucleic acid. A flow cytometer (Attune CytPix) was used for analysis, calibrated and set according to the manufacturer’s instructions. The stained cell suspension was analyzed using the flow cytometer, and the fluorescence signal of PI was detected using the FL2 channel (585/42 nm bandpass filter). At least 10,000 cell events were collected, and the fluorescence intensity distribution for each sample was recorded, followed by data analysis using the accompanying software. Appropriate gating was established to distinguish different phases of the cell cycle (such as G0/G1, S, and G2/M phases). The apoptosis rate was calculated based on the proportion of the sub-G1 peak.

#### 2.3.3 Protein expression in western blot experimental cells

HCC cells were stably transfected during the logarithmic growth phase using standard culture methods. The cells in the culture flask were gently washed with PBS to remove old culture medium and other impurities. The culture flask was then placed on ice for 15 min to facilitate cell detachment. A cell scraper was used to gently detach the cells from the culture surface.

The cell fragments and lysis buffer were transferred to 1.5 mL microcentrifuge tubes. Centrifugation was performed at 4°C, and the supernatant was carefully aspirated while discarding the pellet. To the residual protein extract, one volume of 4× concentrated sample buffer (5×) was added and mixed thoroughly. The mixture was then boiled in water for 10 min to denature the proteins. Following this, the centrifuge was set to 12,000 rpm at room temperature and centrifuged for 5 min. The supernatant was collected and stored at −20°C for future use. The protein concentration of the obtained extracts was quantified using a bicinchoninic acid (BCA) protein assay kit. Based on the protein quantification results, the concentrations of each sample were adjusted to a consistent level, typically 20–30 μg of total protein per well. An appropriate amount of sample buffer was added, mixed, and then boiled again for 5 min. A 10 or 12% sodium dodecyl sulfate-polyacrylamide gel electrophoresis gel was prepared. Samples were loaded into the gel wells, along with pre-stained or unstained protein markers as molecular weight standards. Electrophoresis was performed until the bromophenol blue tracking dye reached the bottom of the gel. The membrane was blocked with 5% non-fat dry milk or BSA in Tris-buffered saline with Tween (TBST) for 1 h to reduce nonspecific binding. Following the dilution ratios specified in the antibody protocols, the membrane was incubated overnight at 4°C with primary antibodies against JAK1, p-JAK1, STAT3, p-STAT3, Bcl-2, Bax, Caspase-9, Caspase-3, and β-actin (as a loading control). The membrane was washed three times with TBST for 5–10 min each time to remove unbound primary antibodies. Afterward, corresponding HRP-conjugated secondary antibodies were added according to the dilution ratios recommended in the antibody protocols, and the membrane was incubated at room temperature for 1 h. The membrane was washed three times with TBST for 5–10 min each time to remove unbound secondary antibodies. The protein concentrations of the obtained samples were quantified using a BCA protein assay kit (Shanghai Fusheng Industrial Co., Shanghai, China). The gray values of each target protein band were measured and normalized to the β-actin band. The relative expression levels among different groups were calculated.

#### 2.3.4 Detection of Caspase-3/9 activity by colorimetric assay

The cells in the culture flask were washed with PBS solution and placed on ice for 15 min for lysis. After the cells in the culture flask were scraped and lysed, the cell debris was further transferred to a 1.5 mL Eppendorf tube with the lysate, and following the instructions on the kit, Caspase-3/9 activities were detected.

### 2.4 Statistical analysis

Data were analyzed using *SPSS version 28.0* statistical software. The Shapiro–Wilk test was employed to assess the normality of the data distribution. Normally distributed data are presented as mean ± standard deviation ( \[\bar{x}]\] ± *s*), while non-normally distributed data are reported as medians (interquartile ranges). For normally distributed data, one-way analysis of variance (ANOVA) was used to compare group differences, followed by *post hoc* comparisons using the least significant difference method. Non-normally distributed data were analyzed using the Kruskal–Wallis rank sum test to compare differences among groups, with a significance threshold set at *P* < 0.05.

## 3 Results

### 3.1 TPL inhibits the proliferation of HCC cells

The cell proliferation inhibition effect in each dose group of TPL was reduced than in the control group, and the inhibition rate of cell proliferation increased with the increase of TPL dose (*P* < 0.05) (Table 1).

**Table 1:** OD values of cell proliferation in different groups ( \[\bar{x}]\] ± *s*)

Group	Dosage (μM)	24 h	48 h	72 h
Control	0	0.32 ± 0.07	0.57 ± 0.04	0.92 ± 0.07
0.02 μM TPL	0.02	0.27 ± 0.05^a^	0.45 ± 0.05^a^	0.58 ± 0.03^a^
0.05 μM TPL	0.05	0.17 ± 0.03^ab^	0.23 ± 0.03^ab^	0.27 ± 0.05^ab^
0.10 μM TPL	0.10	0.05 ± 0.02^abc^	0.07 ± 0.02^abc^	0.09 ± 0.02^abc^
*F*		181.782	109.06	524.56
*P*		<0.001	<0.001	<0.001

Note: *F* refers to the *F* statistic in the ANOVA, \[\bar{x}]\] ± *s* indicates mean ± standard deviation. The notation a denotes significance compared to the control group (*P* < 0.05), b indicates significance compared to the 0.02 μM TPL group (*P* < 0.05), and c signifies significance compared to the 0.05 μM TPL group (*P* < 0.05).

### 3.2 TPL promotes apoptosis in HCC cells

TPL groups had a higher apoptosis rate than those in the control group, and as the TPL dose increased, the apoptosis rate also increased (*P* < 0.05) (Table 2).

**Table 2:** Apoptosis rate among different groups ( \[\bar{x}]\] ± *s*)

Group	Apoptosis rate (%)
Control	2.25 ± 0.36
0.02 μM TPL	6.52 ± 1.65^a^
0.05 μM TPL	11.23 ± 3.63^ab^
0.10 μM TPL	17.58 ± 4.21^abc^
*F*	753.85
*P*	<0.001

Note: *F* refers to the *F* statistic in the ANOVA, \[\bar{x}]\] ± *s* indicates mean ± standard deviation. The notation a denotes significance compared to the control group (*P* < 0.05), b indicates significance compared to the 0.02 μM TPL group (*P* < 0.05), and c signifies significance compared to the 0.05 μM TPL group (*P* < 0.05).

### 3.3 TPL inhibits the expression of p-JAK1 and p-STAT3 proteins

**Figure 1:**
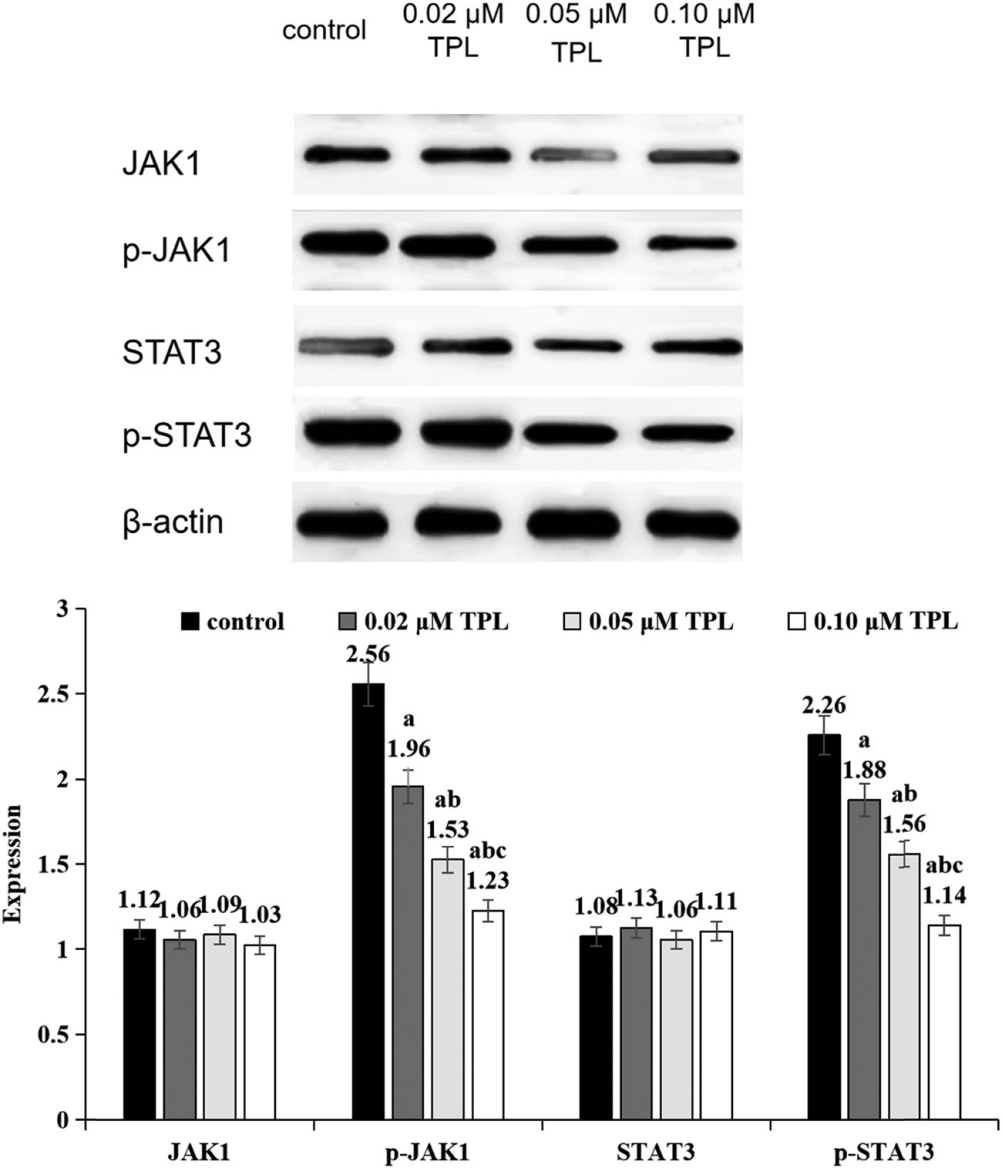
Expression levels of JAK/STAT signaling pathway-related proteins in different groups (a indicates significance compared to the control group, *P* < 0.05; b indicates significance compared to the 0.02 μM TPL group, *P* < 0.05; c indicates significance compared to the 0.05 μM TPL group, *P* < 0.05).

In each dose group of TPL, p-JAK1 and p-STAT3 protein expressions were lower than those in the control group, and decreased with the increase of TPL dose (*P* < 0.05) (Figure 1).

### 3.4 TGF-β1 on the apoptosis rate and JAK/STAT pathway-related protein expression

The expression of P-JAK1 and P-STAT3 proteins in the 0.10 μM TPL + 10 ng/mL TGF-β1 group was lower than that in the 0.10 μM TPL group (*P* < 0.05) (Table 3).

**Table 3:** TGF-β1 on the apoptosis rate and the expression of JAK/STAT pathway-related proteins ( \[\bar{x}]\] ± *s*)

Group	Apoptosis rate (%)	JAK1	p-JAK1	STAT3	p-STAT3
Control	—	1.12 ± 0.12	2.56 ± 0.21	1.08 ± 0.18	2.26 ± 0.24
0.10 μM TPL	17.58 ± 4.21	1.03 ± 0.07	1.23 ± 0.22	1.11 ± 0.13	1.14 ± 0.15
0.10 μM TPL + 10 ng/mL TGF-β1	8.65 ± 2.37	1.08 ± 0.11	1.56 ± 0.17	1.09 ± 0.14	1.67 ± 0.23
*F*	5.563	0.257	2.678	0.135	4.356
*P*	<0.001	0.457	<0.001	0.864	<0.001

Note: *F* refers to the *F* statistic in the ANOVA, \[\bar{x}]\] ± *s* indicates mean ± standard deviation. The notation a denotes significance compared to the control group (*P* < 0.05), b indicates significance compared to the 0.02 μM TPL group (*P* < 0.05), and c signifies significance compared to the 0.05 μM TPL group (*P* < 0.05).

### 3.5 Apoptosis-related protein expression in different groups

**Figure 2:**
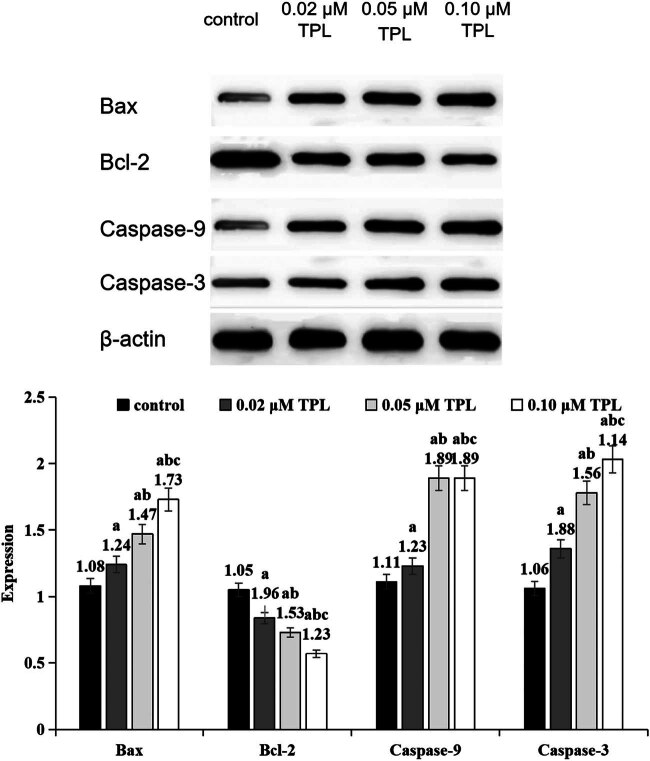
Expression levels of apoptosis-related proteins in different groups (a indicates significance compared to the control group, *P* < 0.05; b indicates significance compared to the 0.02 μM TPL group, *P* < 0.05; c indicates significance compared to the 0.05 μM TPL group, *P* < 0.05.).

In each dose of TPL, Bax and Caspase-9 protein levels were higher than that in the control group, while Bcl-2 and Caspase-3 protein levels were lower. With the increase of TPL dose, the protein of Bax and Caspase-9 increased, while the Bcl-2 and Caspase-3 protein were decreased (*P* < 0.05) (Figure 2).

## 4 Discussion

TPL, a diterpene tricyclic oxide purified from *T. wilfordii* Hook F, a medicinal plant used to treat inflammatory diseases, and is effective in inhibiting a variety of cancer cell lines, including HCC and colorectal cancer [9]. TPL inhibits mRNA transcription by targeting transcription factors XPB and RNA Pol II. RNA polymerase II’s largest subunit, Rpb1, is degraded by TPL. At the same time, TPL destroys nucleolar integrity and inhibits ribosomal RNA transcription mediated by Pol I. Therefore, TPL can be used as an inhibitor of Pol I and Pol II [10]. In related studies, TPL inhibits the HCCMHCC-97H cells invasion and tumorigenesis [11]. Some studies noted that TPL significantly inhibits miR-17-92 and miR-106b-25, leading to the upregulation of their common target genes, BIM, PTEN, and p21. c-Myc is necessary for the inhibition of these miRNAs in HCC samples, resulting in increased cell death induced by TPL. Additionally, TPL downregulates c-Myc expression by targeting ERCC3, a protein that binds to TPL. The regulation of the c-Myc/miRNA cluster/target gene axis by TPL enhances its antitumor activity, suggesting that TPL could be an effective anticancer agent for liver cancer [12]. In this study, the inhibition rate of cell proliferation in each TPL dose group was lower than that in the control group, and this effect depended on the increased dosage of TPL, consistent with previous studies.

There is an important role for JAK/STAT signaling in cell proliferation, stem cell maintenance and differentiation, and regulation of immune/inflammatory response [13]. It is reported that JAK/STAT signal transduction can regulate gluconeogenesis and liver regeneration [14]. JAK is activated during ligand-induced conformational changes of the receptor, establishing docking sites for transcriptional activator family (STAT). The STAT protein is phosphorylated by JAK, activated to form a dimer, and then transferred to the nucleus after binding to the receptor. Consequently, the STAT dimer recognizes and binds specific promoter sequences to activate transcription of its target genes, CCND1, BIRC5, and Mcl-1 [15]. Studies have shown that the JAK/STAT pathway is usually altered in cancer, including HCC. In up to 60% of cases of HCC, STAT3 is constitutively active. JAK/STAT signal is upregulated by inflammation, and epigenetic silencing of SOCS gene [16]. Abnormal activation contributes to the development and progress of HCC [17]. The progression of the cell cycle from the G1 phase to the S phase is accelerated in cells with constitutively activated STAT3. An inhibition of the JAK2/STAT3 pathway could significantly suppress CCND1 expression and arrest the growth of HCC cells in G0/G1 [18]. The introduction of STAT3-specific short hairpin RNA into diethylnitrosamine-induced HCC mouse models can inhibit tumor progression as well, indicating the carcinogenic role of STAT3 in HCC. This study found that the expression levels of p-JAK1 and p-STAT3 proteins in all TPL dose groups were lower than those in the control group, and the expression of p-JAK1 and p-STAT3 decreased with increasing TPL dosage. This indicates that TPL exerts a significant dose-dependent inhibitory effect on the expression of p-JAK1 and p-STAT3 in HCC cells. Specifically, as the concentration of TPL increases, the phosphorylation levels of p-JAK1 and p-STAT3 gradually decrease, suggesting that TPL effectively inhibits the activation of the JAK/STAT signaling pathway. These results imply that TPL may interfere with the phosphorylation processes of JAK1 and STAT3, thereby blocking downstream signal transduction in this pathway, which in turn suppresses tumor cell proliferation and promotes apoptosis. This dose-dependent inhibitory effect further supports the feasibility of TPL as a potential anticancer drug. Previous studies have also reported similar findings, demonstrating that TPL inhibits the JAK/STAT pathway in various cancer models and exhibits robust anticancer activity in both *in vivo* and *in vitro* experiments [19]. Our findings are consistent with these previous results, further confirming the potential of TPL in the treatment of HCC and providing important theoretical support for the future development of novel anticancer strategies based on the JAK/STAT pathway.

In the high-dose TPL group, the addition of transforming growth factor-beta (TGF-β) reduced apoptosis and increased the expression of p-JAK1 and p-STAT3, revealing a complex interaction between TPL and TGF-β. TGF-β is a multifunctional cytokine that typically exerts anti-cancer effects in the early stages but may promote tumor progression in certain contexts. By activating the JAK/STAT signaling pathway, TGF-β may counteract the inhibitory effects of TPL on these pathways, thereby diminishing TPL-induced apoptosis. This interaction suggests that TGF-β may protect cells from TPL-induced apoptosis by upregulating the JAK/STAT signaling pathway. This finding has significant implications for the therapeutic use of TPL, indicating that the levels of TGF-β and its impact on signaling pathways should be considered in clinical applications. Furthermore, it suggests that combining TPL with other agents that can inhibit TGF-β or the JAK/STAT pathway may enhance its anticancer effects, providing new directions for the development of more effective treatment strategies. Further investigation into this interaction will contribute to optimizing TPL dosing regimens and improving its efficacy in the treatment of HCC and other cancers.

This study found that TPL not only exerts a significant dose-dependent inhibitory effect on the JAK1/STAT3 signaling pathway but also promotes apoptosis in HCC cells by regulating the Bax/Bcl-2 ratio and activating Caspase-3/9. Specifically, as the concentration of TPL increases, the expression level of the pro-apoptotic protein Bax rises, while the expression level of the anti-apoptotic protein Bcl-2 decreases, leading to a significant increase in the Bax/Bcl-2 ratio. This change disrupts mitochondrial membrane stability, facilitating the release of cytochrome c into the cytoplasm, which subsequently activates Caspase-9 and triggers the activation of downstream effector Caspase-3, ultimately inducing apoptosis. These results indicate that TPL promotes programmed cell death in HCC cells by regulating the Bax/Bcl-2 ratio and activating the Caspase cascade. This mechanism aligns with the classical mitochondrial apoptosis pathway, further supporting TPL’s role as a potential anticancer drug and providing a solid biological foundation for its application in HCC treatment. Previous studies have also reported similar findings, demonstrating that TPL can induce apoptosis in various cancer cells by affecting Bcl-2 family proteins and Caspase activity [20]. Our results are consistent with these findings, further validating the multifunctionality and broad applicability of TPL in cancer therapy.

To sum up, TPL can effectively inhibit hepatoma cell proliferation and promote cancer cell apoptosis, and exert its anti-hepatoma effect, and inhibiting JAK1/STAT3 signaling may activate apoptosis-related pathways.

## 5 Conclusion

This study systematically evaluated the effects of TPL on the proliferation and apoptosis of HepG2 liver cancer cells, revealing a significant dose-dependent effect of TPL in inhibiting cell proliferation and promoting apoptosis. The results showed that the cell proliferation inhibition rate in the TPL treatment groups increased with higher dosages, while the apoptosis rate also rose significantly. Additionally, TPL upregulated the activity of the pro-apoptotic proteins Bax and Caspase-3/9, while downregulating the expression of the anti-apoptotic protein Bcl-2, further confirming its role in inducing cell death via the mitochondrial apoptosis pathway. In the high-dose TPL group, the addition of TGF-β resulted in reduced apoptosis and increased expression of p-JAK1 and p-STAT3 proteins, suggesting that TGF-β may partially counteract the anticancer effects of TPL by activating the JAK/STAT pathway. These findings not only highlight the efficacy and multifunctionality of TPL as a potential anti-liver cancer drug but also elucidate its mechanisms involving the inhibition of the JAK1/STAT3 signaling pathway and the activation of apoptosis-related pathways. Future studies should further explore the interactions between TPL and other signaling pathways, considering the combination of TPL with other targeted therapies to optimize treatment regimens. Furthermore, these results provide important theoretical support for the development of novel anticancer strategies based on the JAK/STAT pathway and offer new directions for clinical applications.

## References

[1] Raymant M, Astuti Y, Alvaro-Espinosa L, Green D, Quaranta V, Bellomo G, et al. Macrophage-fibroblast JAK/STAT dependent crosstalk promotes liver metastatic outgrowth in pancreatic cancer. Nat Commun. 2024;15(1):3593. 10.1038/s41467-024-47949-3 PMC11055860 38678021

[2] Liu J, Wang FP, Luo FM. The role of JAK/STAT pathway in fibrotic diseases: molecular and cellular mechanisms. Biomolecules. 2023;13(1):119. 10.3390/biom13010119 PMC9855819 36671504

[3] Jia JJ, Zhou XL, Chu QF. Mechanisms and therapeutic prospect of the JAK-STAT signaling pathway in liver cancer. Mol Cell Biochem. 2024. 10.1007/s11010-024-04983-5. 38519710

[4] Hu WM, Liu SQ, Zhu KF, Li W, Yang ZJ, Yang Q, et al. The ALOX5 inhibitor Zileuton regulates tumor-associated macrophage M2 polarization by JAK/STAT and inhibits pancreatic cancer invasion and metastasis. Int Immunopharmacol. 2023;121:110505. 10.1016/j.intimp.2023.110505 37348233

[5] Park H, Lee S, Lee J, Moon H, Ro SW. Exploring the JAK/STAT signaling pathway in hepatocellular carcinoma: unraveling signaling complexity and therapeutic implications. Int J Mol Sci. 2023;24(18):13764. 10.3390/ijms241813764 PMC10531214 37762066

[6] Rah B, Farhat NM, Hamad M, Muhammad JS. JAK/STAT signaling and cellular iron metabolism in hepatocellular carcinoma: therapeutic implications. Clin Exp Med. 2023;23(7):3147–57. 10.1007/s10238-023-01047-8 36976378

[7] Reece MD, Song C, Hancock SC, Pereira Ribeiro S, Kulpa DA, Gavegnano C. Repurposing BCL-2 and Jak 1/2 inhibitors: cure and treatment of HIV-1 and other viral infections. Front Immunol. 2022;13:1033672. 10.3389/fimmu.2022.1033672 PMC9782439 36569952

[8] Yuan L, Cai YQ, Zhang L, Liu SJ, Li P, Li XL. Promoting apoptosis, a promising way to treat breast cancer with natural products: a comprehensive review. Front Pharmacol. 2022;12:801662. 10.3389/fphar.2021.801662 PMC8836889 35153757

[9] Liao SX, Deng JW, Deng ML, Chen CY, Han FY, Ye KH, et al. AFDN deficiency promotes liver tropism of metastatic colorectal cancer. Cancer Res. 2024;84(19):3158–72. 10.1158/0008-5472.CAN-23-3140 39047222

[10] Sethi G, Rath P, Chauhan A, Ranjan A, Choudhary R, Ramniwas S, et al. Apoptotic mechanisms of quercetin in liver cancer: recent trends and advancements. Pharmaceutics. 2023;15(2):712. 10.3390/pharmaceutics15020712 PMC9960374 36840034

[11] Chaker D, Desterke C, Moniaux N, Bani MA, Oudrhiri N, Faivre J, et al. Direct reprogramming of hepatocytes into JAK/Stat-dependent LGR5 + liver cells able to initiate intrahepatic cholangiocarcinoma. Stem Cell. 2024;42(4):301–16. 10.1093/stmcls/sxae006 38262709

[12] Wang YY, Lai WZ, Zheng XJ, Li K, Zhang YH, Pang XJ, et al. Linderae Radix extract attenuates ulcerative colitis by inhibiting the JAK/STAT signaling pathway. Phytomedicine. 2024;132:155868. 10.1016/j.phymed.2024.155868 39032278

[13] Wuerger LTD, Kudiabor F, Alarcan J, Templin M, Poetz O, Sieg H, et al. Okadaic acid activates JAK/STAT signaling to affect xenobiotic metabolism in HepaRG cells. Cells. 2023;12(5):770. 10.3390/cells12050770 PMC10000888 36899906

[14] Zhang M, Lai JM, Wu QL, Lai J, Su JY, Zhu B, et al. Naringenin induces HepG2 cell apoptosis via ROS-mediated JAK-2/STAT-3 signaling pathways. Molecules. 2023;28(11):4506. 10.3390/molecules28114506 PMC10254813 37298981

[15] Rahimi S, van Leeuwen D, Roshanzamir F, Pandit S, Shi L, Sasanian N, et al. Ginsenoside Rg3 reduces the toxicity of graphene oxide used for pH-responsive delivery of doxorubicin to liver and breast cancer cells. Pharmaceutics. 2023;15(2):391. 10.3390/pharmaceutics15020391 PMC9965446 36839713

[16] Lin LL, Chen Q. Yadanziolide a inhibits proliferation and induces apoptosis of hepatocellular carcinoma via JAK-STAT pathway: a preclinical study. Biology (Basel). 2024;13(7):528. 10.3390/biology13070528 PMC11274273 39056720

[17] Subbarayan R, Srinivasan D, Shadula Osmania S, Murugan Girija D, Ikhlas S, Srivastav N, et al. Molecular insights on Eltrombopag: potential mitogen stimulants, angiogenesis, and therapeutic radioprotectant through TPO-R activation. Platelets. 2024;35(1):2359028. 10.1080/09537104.2024.2359028 38832545

[18] Liu XQ, Pang XQ, Wan ZP, Zhao JH, Gao ZL, Deng H. Dopamine inhibits the expression of hepatitis B virus surface and e antigens by activating the JAK/STAT pathway and upregulating interferon-stimulated gene 15 expression. J Clin Transl Hepatol. 2024;12(5):443–56. 10.14218/JCTH.2024.00051 PMC11106351 38779516

[19] Wu JB, Sun M, Li ZZ, Shen Y, Wu YJ, Zhang HS, et al. Effect of urokinase-type plasminogen activator combined with clinical stage and Barcelona Clinic Liver Cancer stage on the prognosis of patients with hepatocellular carcinoma. J Gastrointest Oncol. 2023;14(3):1434–50. 10.21037/jgo-23-311 PMC10331755 37435232

[20] Zhang LH, Zhang W, Chen JL. Regulatory mechanism of immune-related genes in patients with hypertension. Medicine (Baltimore). 2023;102(9):e32627. 10.1097/MD.0000000000032627 PMC9981365 36862882

